# Higher brain extracellular potassium is associated with brain metabolic distress and poor outcome after aneurysmal subarachnoid hemorrhage

**DOI:** 10.1186/cc13916

**Published:** 2014-06-11

**Authors:** Ana Patrícia Antunes, Alois Josef Schiefecker, Ronny Beer, Bettina Pfausler, Florian Sohm, Marlene Fischer, Anelia Dietmann, Peter Lackner, Werner Oskar Hackl, Jean-Pierre Ndayisaba, Claudius Thomé, Erich Schmutzhard, Raimund Helbok

**Affiliations:** 1 Neurological Intensive Care Unit, Department of Neurology, Innsbruck Medical University, Innsbruck, Austria; 2 Department of Neurosciences, North Lisbon Hospital Centre, Santa Maria Hospital, Lisbon, Portugal; 3 Department of Neurosurgery, Innsbruck Medical University, Innsbruck, Austria; 4 Institute of Biomedical Informatics, UMIT–University for Health Sciences, Medical Informatics and Technology, Eduard Wallnoefer-Zentrum 1, 6060 Hall, Austria

## Abstract

**Introduction:**

Elevated brain potassium levels ([K^+^]) are associated with neuronal damage in experimental models. The role of brain extracellular [K^+^] in patients with poor-grade aneurysmal subarachnoid hemorrhage (aSAH) and its association with hemorrhage load, metabolic dysfunction and outcome has not been studied so far.

**Methods:**

Cerebral microdialysis (CMD) samples from 28 poor grade aSAH patients were analyzed for CMD [K^+^] for 12 consecutive days after ictus, and time-matched to brain metabolic and hemodynamic parameters as well as corresponding plasma [K^+^]. Statistical analysis was performed using a generalized estimating equation with an autoregressive function to handle repeated observations of an individual patient.

**Results:**

CMD [K^+^] did not correlate with plasma [K^+^] (Spearman’s ρ = 0.114, *P* = 0.109). Higher CMD [K^+^] was associated with the presence of intracerebral hematoma on admission head computed tomography, CMD lactate/pyruvate ratio >40 and CMD lactate >4 mmol/L (*P* < 0.05). *In vitro* retrodialysis data suggest that high CMD [K^+^] was of brain cellular origin. Higher CMD [K^+^] was significantly associated with poor 3-month outcome, even after adjusting for age and disease severity (*P* < 0.01).

**Conclusions:**

The results of this pilot study suggest that brain extracellular [K^+^] may serve as a biomarker for brain tissue injury in poor-grade aSAH patients. Further studies are needed to elucidate the relevance of brain interstitial K^+^ levels in the pathophysiology of secondary brain injury after aSAH.

## Introduction

Maintenance of brain ion homeostasis is critical for synaptic transmission and proper functioning of the neuron-glia signalling network [1]. After aneurysmal subarachnoid hemorrhage (aSAH), several mechanisms may contribute to transient or prolonged accumulation of brain extracellular potassium (K^+^): erythrocytolysis [2], unspecific membrane breakdown due to parenchymal injury [3-5], dysfunction of Na^+^/K^+^ pump [1,6-8] and, on the one hand, compromised glial buffering [9,10], on the other hand, activation of neuronal ATP–sensitive, or G protein–dependent, calcium-sensitive K^+^ channels due to energy failure [11,12]. During aSAH, both blood compounds [3,13] and ischemia [14] may induce cortical spreading depolarization (CSD). CSD leads to a transient or sustained increase of brain extracellular K^+^ concentration ([K^+^]) up to 60 mmol/L, far beyond its physiological range [8]. Notably, ictal epileptic events and spreading depolarization are the only events in the brain during which extracellular [K^+^] reaches such high levels [12,14]. For comparison, during ictal epileptic events, extracellular [K^+^] increases from only 3 to 12 mmol/L [15].

In patients with severe traumatic brain injury (TBI), higher brain extracellular [K^+^] was correlated with higher cerebral microdialysis (CMD) lactate and CMD glutamate levels, increased intracranial pressure (ICP) and poor functional outcome, thus supporting a relationship between higher brain extracellular [K^+^] and dysfunction and/or damage of the brain parenchyma [5]. Derangement of plasma [K^+^] is associated with muscle weakness and cardiac arrhythmia as well as, to a lesser degree, cerebral symptoms such as lethargy, irritability, confusion and coma. Neurological symptoms are more likely related to concomitant Na^+^ and acid-base disturbances, suggesting that brain extracellular [K^+^] may be independent of plasma K^+^ levels. The results of studies done in animal models have shown that the disrupted blood–brain barrier can be open to large molecules, but can maintain the stability of the extracellular [K^+^] [16]. Little is known about the relationship between plasma and cerebral extracellular [K^+^] after acute brain injury in humans.

In this pilot study, we investigated the relationship between brain extracellular [K^+^] and plasma [K^+^] and the association of high CMD [K^+^] with brain metabolic and hemodynamic parameters and functional outcome in poor-grade aSAH patients.

## Material and methods

### Study population

Data were recorded prospectively for 28 consecutive aSAH patients who underwent brain multimodal neuromonitoring, including ICP, brain tissue oxygen tension (P_bt_O_2_) and cerebral microdialysis, between September 2010 and October 2012, and the CMD samples were retrospectively analyzed for CMD [K^+^]. The following institutional criteria for invasive neuromonitoring were applied: (1) Glasgow Coma Scale (GCS) score ≤8 upon admission or deterioration to a GCS score ≤8 during hospitalization, (2) low likelihood of regaining consciousness within the next 48 hours, (3) high likelihood of surviving at least 48 hours and (4) age > = 18 years. The study was approved by the Independent Ethics Committee of Innsbruck Medical University (UN3898 285/4.8) and was carried out in adherence to the ethical conduct of clinical research involving critically ill patients issued by the Austrian Federal Office for Safety in Health Care. In line with this, if incapacity as a result of the critical illness precluded standard informed consent, deferred consent was required according to the Independent Ethics Committee approval. After regaining the ability to consent, patients were informed without delay and asked for their consent for further participation in the study. In the case of persistent incapacity, proxy consent was obtained according to Austrian law. Additionally, the public on the research site was informed about the study by notice on the bulletin board at the neurological ICU.

### General management

Patient care was carried out in adherence to the guidelines of the American Heart Association for management of aSAH [17]. Hemodynamic and fluid management were targeted to achieve a cerebral perfusion pressure (CPP) >70 mmHg. The target for intracranial pressure (ICP) was <20 mmHg. Patients were treated to maintain euvolemia, normonatremia and normokalemia, depending on disease severity and left at the discretion of the attending neurintensivist.

### Cerebral microdialysate and plasma potassium measurements

Three consecutive CMD samples were pooled to obtain >40 μl for analysis of CMD [K^+^] using the i-STAT system and i-STAT EC8*+* cartridges (Abbott Point of Care, Princeton, NJ, USA) [18]. Plasma electrolytes were measured using the ABL800 FLEX blood gas analyzer (Radiometer Medical, Brønshøj, Denmark).

### Monitoring and data acquisition

Neuromonitoring probes were placed, through a subcutaneous tunnel or using a triple-lumen bolt, into the white matter at greatest risk for secondary brain injury, which we defined as brain tissue with the highest probability for damage due to delayed cerebral infarction. The location of the microdialysis catheter was confirmed by head CT scan, usually obtained within 24 hours after surgery, and graded to distinguish radiologically “normal” from “perilesional” tissue (CMD probe <1 cm distant from a hematoma or hypodensity) or “intralesional” tissue (within a hemorrhagic or ischemic lesion). CMD was performed using a 100 kDa cutoff microdialysis catheter (CMA-71; M Dialysis, Stockholm, Sweden), and perfusion fluid (Na^+^ 147 mmol/L, CaCl^2+^ 1.2 mmol/L, MgCl^2+^ 0.9 mmol/L, KCl^+^ 2.7 mmol/L (CNS perfusion fluid); M Dialysis) was pumped at a flow rate of 0.3 μl/min. Hourly samples were analyzed (CMA 600 and ISCUS ^flex^, M Dialysis AB, Stockholm, Sweden) for interstitial glucose, pyruvate, lactate and glutamate concentrations and frozen at -80°C. ICP was measured with an intraparenchymal probe (NEUROVENT; RAUMEDIC, Helmbrechts, Germany), and P_bt_O_2_ was measured using a Clark-type probe (LICOX; Integra LifeSciences, Plainsboro, NJ, USA). In weekly meetings of the study team (RH, BP, RB, MF, AS and ES), disease and treatment complications were evaluated as part of the development of an ongoing prospective database. Delayed cerebral infarction was defined as a newly appearing infarction found on follow-up brain CT scans and judged by an independent radiologist to be attributable to vasospasm. Pneumonia was defined as chest X-ray infiltrate plus elevated systemic inflammatory parameters. Survival and functional outcome were evaluated prospectively with the modified Rankin Scale (mRS) 3 months after aSAH by telephone interview conducted by a study nurse blinded to neuromonitoring data. Poor neurological outcome was defined as mRS score >4 (severe disability or death). An electronic patient data management system (Centricity Clinical Notification System with Centricity Critical Care 7.0 software; GE Healthcare, Little Chalfont, UK) was used to acquire digital data for blood pressure, ICP, CPP and P_bt_O_2_ every 3 minutes from the monitoring device (CARESCAPE Monitor B650; GE Healthcare). Brain tissue hypoxia was defined as P_bt_O_2_ < 20 mmHg, based on previous studies demonstrating a higher risk of metabolic distress and poor outcome below this threshold value [19,20]. Low CPP was considered ≤70 mmHg in accordance with data showing an association of this threshold with metabolic crisis (CMD lactate/pyruvate ratio (LPR) >40 and CMD glucose <0.7 mmol/L) and brain tissue hypoxia [19]. Metabolic distress was defined as CMD LPR >40. We also categorized data for CMD LPR >25, deeming it an early warning sign of metabolic distress [21,22]. High CMD lactate, high CMD glutamate and low CMD pyruvate were considered to be present when measurements >4 mmol/L [20,21], >10 μmol/L [22] and <119 μmol/L [20,23], respectively, were observed.

### Statistical analysis

Data for CPP, ICP, P_bt_O_2_, plasma [K^+^] and CMD metabolites were time-matched to the 3-hour period of the pooled sample and averaged to the corresponding CMD [K^+^] analysis. Continuous variables were dichotomized based on clinical cutoff points or median values. Univariate and multivariate comparisons of pooled data were performed using a generalized linear model with a binomial distribution and logit link function. The model was extended by using generalized estimating equations with the autoregressive matrix of the first order to handle repeated observations of the same patient [24].

The percentage of CMD samples with CMD [K^+^] above the median was calculated for each patient, to assess differences between outcome groups (Mann–Whitney *U* test). For correlation analyses, Spearman’s ρ was used. The results are reported as medians with IQRs unless otherwise specified. *P* < 0.05 was considered statistically significant. SPSS 21 software (SPSS Inc, Chicago, IL, USA) was used for data analysis.

### *In vitro* potassium measurements

*In vitro* analysis was performed using a 100 kDa cutoff CMD catheter (CMA-71) at a constant flow rate of 0.3 μl/min to determine the relative recovery rate. The relative recovery rate was defined as the [K^+^] level in the microdialysate divided by [K^+^] in three different K^+^ dilutions (low, medium and high). The K^+^ dilution was created using concentrated K^+^ (1 mmol/ml K^+^) diluted by isotonic NaCl to obtain low (2.2 mmol/L), medium (4.3 mmol/L) and high (9.0 mmol/L) K^+^ dilutions. The tip of the CMD catheter was then inserted into the K^+^ dilution. Microdialysate was collected hourly and pooled over the course of 3 hours for analysis using the method described above. [K^+^] in the microdialysate was 9.0 ± 0 mmol/L (SD) in the high [K^+^] dilution, 4.2 ± 0.07 mmol/L (SD) in the medium [K^+^] dilution and 2.0 ± 0.14 mmol/L (SD) in the low [K^+^] dilution after repetitive analyses, revealing relative *in vitro* recovery rates of 100% in the presence of high [K^+^], 98% in the presence of medium [K^+^] and 91% in the presence of low [K^+^]. In order to determine the accuracy of this method, we double-analyzed the same [K^+^] dilutions at 3 hours, 6 hours and 9 hours after the beginning of *in vitro* microdialysis, which revealed identical [K^+^] at all time points.

## Results

### Study population

Twenty-eight aSAH patients were included in our analysis (Table 1). Neuromonitoring was initiated on day 1 after ictus (1 to 2 days). The CMD catheter location was in radiologically normal tissue in 15 patients (54%) and in perilesional tissue in 13 patients (46%). Four patients died during hospitalization due to brain herniation (*n* = 2), mesenteric ischemia (*n* = 1) or septic shock (*n* = 1). One of these patients died during the neuromonitoring period on the tenth day after ictus.

**Table 1:** **Patient characteristics (*N*** **= 28)**^**a**^

**Clinical characteristics**	**Data**
Age (yr)	56 (47 to 68)
Sex (female)	16 (57%)
Admission H&H grade	
2	3 (11%)
3	6 (21%)
4	3 (11%)
5	16 (57%)
Admission APACHE II score	18 (14 to 21)
Admission radiological characteristics	
Modified Fisher scale score	
1	3 (11%)
2	3 (11%)
3	10 (35%)
4	12 (43%)
IVH sum score	5 (0 to 9)
Intracerebral hematoma	13 (46%)
Aneurysm size >10 mm	8 (29%)
Generalized cerebral edema	11 (39%)
Surgical procedures/interventions	
Clipping	20 (71%)
Hydrocephalus requiring CSF diversion	22 (79%)
Hyperosmolar therapy	14 (50%)
Complications	
Pneumonia	21 (75%)
Delayed cerebral infarction	6 (21%)
Outcome characteristics	
Length of hospital stay (days)	40 (27 to 50)
3-month mRS score	
0 to 1	5 (17.9%)
2 to 3	6 (21.4%)
4	6 (21.4%)
5	6 (21.4%)
6	5 (17.9%)

^a^APACHE II = Acute Physiology and Chronic Health Evaluation II; CSF = Cerebrospinal fluid; H&H = Hunt and Hess; IVH = Intraventricular hemorrhage; mRS *=* modified Rankin Scale. Data shown are *n* (%) for categorical variables and median (IQR) for continuous variables.

### Cerebral microdialysate and plasma potassium concentrations

**Figure 1:**
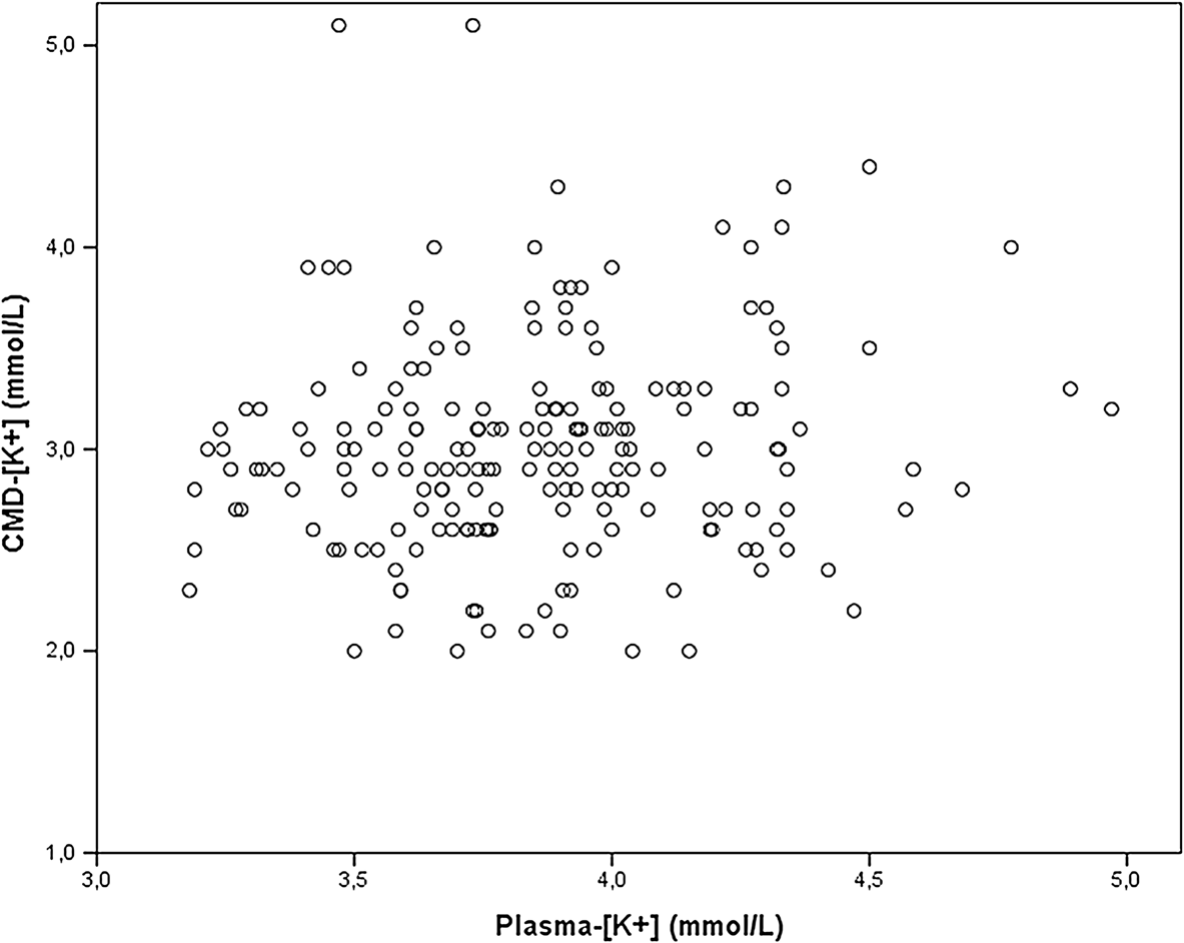
**Correlation between potassium concentrations in plasma and cerebral microdialysate concentrations.** [K^+^] = Potassium concentration; CMD = Cerebral microdialysate.

Admission plasma [K^+^] was 3.7 mmol/L (3.4 to 4 mmol/L) and increased only slightly during hospitalization to 3.9 mmol/L (3.7 to 4.1 mmol/L). Median CMD [K^+^] was 3 mmol/L (2.7 to 3.2 mmol/L). Plasma [K^+^] did not correlate with CMD [K^+^] (Spearman’s ρ = 0.114; *P* = 0.109) (Figure 1).

### Cerebral microdialysate, plasma potassium and brain homeostasis

CMD [K^+^] was positively correlated with CMD lactate levels (Spearman’s ρ 0.453; *P* < 0.0001), CMD LPR (Spearman’s ρ = 0.368; *P* < 0.0001) and CMD glutamate levels (Spearman’s ρ = 0.355; *P* = 0.0002), whereas the strongest correlation was found during days 7 to 12 (CMD lactate: Spearman’s ρ = 0.489, *P* < 0.0001; CMD LPR ρ = 0.006; Spearman’s ρ = 0.463; CMD glutamate ρ = 0.000; Spearman’s ρ = 0.481) and in patients with delayed cerebral infarction (CMD glutamate ρ = 0.005; Spearman’s ρ = 0.840, *P* < 0.01; CMD lactate ρ = 0.003 Spearman’s ρ = 0.773, *P* < 0.01). Univariate analysis revealed an association between higher CMD [K^+^] and CMD LPR >25, CMD LPR > 40, CMD lactate >4 mmol/L, intraventricular hemorrhage (IVH) sum score >5 (median), presence of intracerebral hematoma (ICH) on admission head CT and perilesional probe location (Table 2). In the multivariate model, higher CMD [K^+^] was significantly associated with CMD LPR >25, CMD LPR >40, CMD lactate >4 mmol/L and presence of ICH after adjusting for probe location and CPP ≤70 mmHg (Table 3).

**Table 2:** **Factors associated with higher potassium concentration in cerebral microdialysate**^**a**^

	**CMD [K**^**+**^**] >3 mmol/L**	
**Independent variables**	**OR (95% CI)**	***P*-value**
Age > 56 yr (median)	0.7 (0.3 to 2.0)	0.548
Hunt and Hess grade >3	2.1 (0.8 to 5.5)	0.141
APACHE II score > 18 (median)	1.6 (0.6 to 4.4)	0.322
Perilesional CMD probe location	3.4 (1.3 to 8.9)	0.010
Modified Fisher scale = 4	1.4 (0.5 to 3.8)	0.487
IVH sum score >5 (median)	3.0 (1.1 to 8.1)	0.026
Intracerebral hematoma	3.1 (1.2 to 8.1)	0.022
Bicaudate index >0.2	1.2 (0.4 to 3.4)	0.764
Admission GCE	0.6 (0.2 to 1.9)	0.425
Delayed cerebral infarction	1.4 (0.4 to 5.4)	0.623
ICP >20 mmHg	2.8 (1.0 to 8)	0.058
CPP ≤70 mmHg	1.8 (0.8 to 3.8)	0.128
P_bt_O_2_ < 20 mmHg	1.7 (0.6 to 2.9)	0.422
CMD lactate >4 mmol/L	4.2 (2.0 to 8.7)	0.0001
CMD pyruvate <119 μmol/L	0.7 (0.3 to 1.4)	0.267
CMD LPR >25	3.6 (1.4 to 9.2)	0.008
CMD LPR >40	5.1 (2.5 to 10.1)	<0.0001
CMD glucose <0.7 mmol/L	0.9 (0.5 to 1.7)	0.719
CMD glutamate >10 μmol/L	2.1 (0.9 to 5.0)	0.099

^a^APACHE II = Acute Physiology and Chronic Health Evaluation II; CI = Confidence interval; CMD = Cerebral microdialysate; CPP = Cerebral perfusion pressure; GCE = Generalized cerebral edema; ICP = Intracranial pressure; IVH *=* Intraventricular hemorrhage; [K^+^] = potassium concentration; LPR = Lactate/pyruvate ratio; OR = Odds ratio; P_bt_O_2_ = Brain tissue oxygen tension. Higher CMD [K^+^] was defined as values above the median.

**Table 3:** **Multivariate model of factors associated with higher potassium concentration in cerebral microdialysate**^**a**^

	**CMD [K**^**+**^**] >3 mmol/L**	
**Independent variables**	**OR (95% CI)**	***P*-values**
IVH sum score >5 (median)	2.2 (0.8 to 6.2)	0.124
Intracerebral hematoma	3.3 (1.3 to 8.2)	0.013
ICP >20 mmHg	2.7 (1.0 to 7.3)	0.058
CMD lactate >4 mmol/L	4.3 (2.1 to 8.7)	<0.0001
CMD LPR >25	5.2 (1.8 to 15.0)	0.002
CMD LPR >40	4.4 (2.2 to 8.8)	<0.0001

^a^CI = Confidence interval; CMD = Cerebral microdialysate; ICP = Intracranial pressure; IVH = Intraventricular hemorrhage; LPR = Lactate/pyruvate ratio; OR = Odds ratio. Each model was adjusted for probe location and cerebral perfusion pressure ≤70 mmHg. Higher CMD [K+] was defined as value above the overall median.

There was no association between brain metabolic and physiological parameters and higher plasma [K^+^] (data not shown).

### Cerebral microdialysate potassium concentration, brain metabolism and outcome

**Figure 2:**
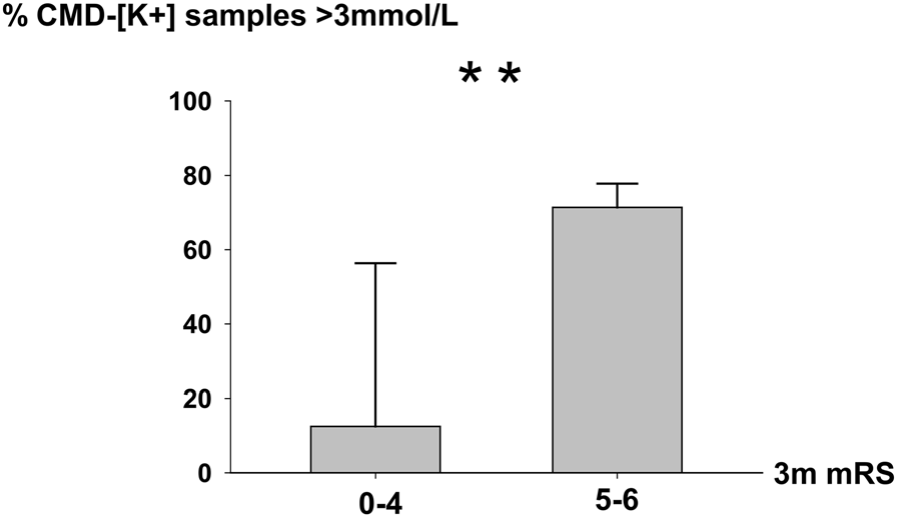
**Potassium concentration in cerebral microdialysate depending on functional outcome.** Percentage of episodes of potassium concentration ([K^+^]) above 3 mmol/L in cerebral microdialysate (CMD) between patients with modified Rankin Scale (mRS) scores ≤4 and those with mRS scores >4 three months after ictus. Bars represent median and IQR. **P = 0.006.

**Figure 3:**
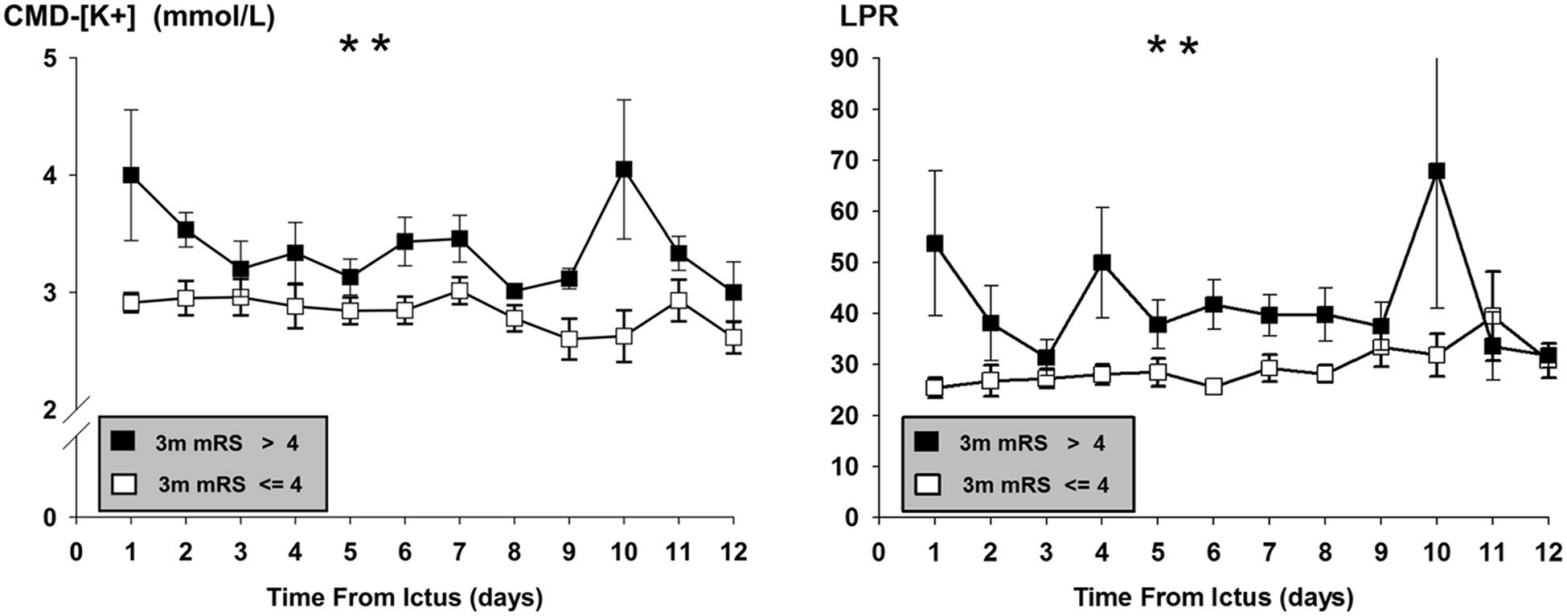
**Daily potassium concentrations and lactate/pyruvate ratio in cerebral microdialysate and functional outcome.** Daily potassium concentration ([K^+^]) and lactate/pyruvate ratio in cerebral microdialysate (CMD) of 28 aneurysmal subarachnoid hemorrhage patients stratified by 3-month modified Rankin Scale scores (3 m mRS) ≤4 (open boxes) and >4 (filled boxes) (mean ± SEM). **P < 0.01. In patients with poor functional outcome, there is a peak CMD [K^+^] on the first day, and, later, on the tenth day after ictus. This evolution of CMD [K^+^] further supports the relationship between brain extracellular (EC) K^+^ levels and early and secondary brain tissue injury.

The percentage of episodes of higher CMD [K^+^] (above the median of 3 mmol/L) was higher in patients with poor 3-month outcomes (71% (50% to 78%)) compared to patients with mRS scores ≤4 (12.5% (0 to 56%)) (*P* = 0.006) (Figure 2). To verify the association between poor outcome and higher CMD [K^+^] and CMD LPR >40, generalized estimation equation models were calculated with day after SAH as the factor and age and admission Hunt and Hess grade as the covariates. These adjusted models showed a statistically significant effect of higher CMD [K^+^] and CMD LPR >40 on poor functional 3-month outcomes (*P* < 0.01) (Figure 3).

## Discussion

**Figure 4:**
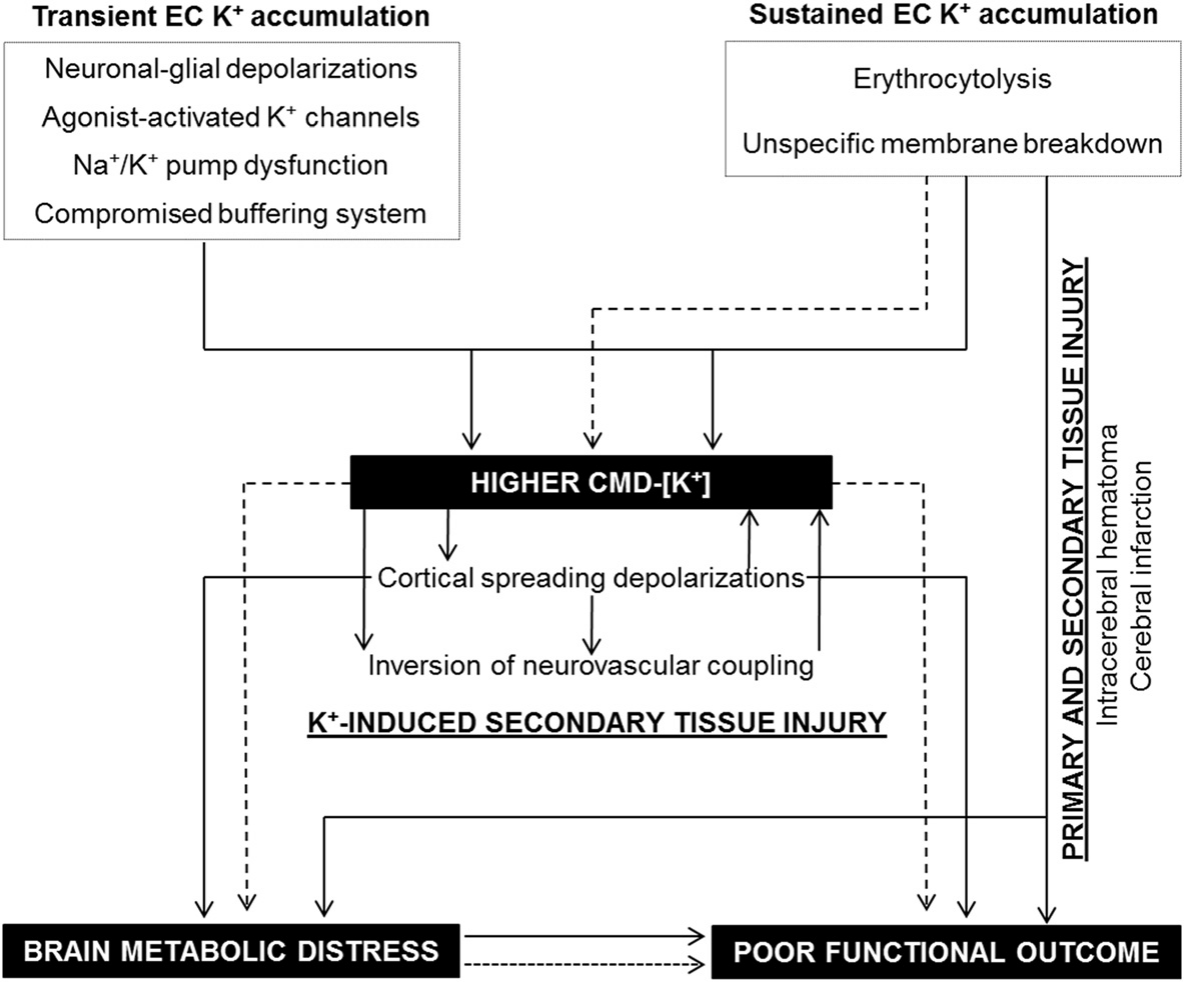
**Interaction diagram between potential causes for and consequences of cerebral extracellular potassium accumulation.** Interaction diagram between brain events that may lead to cerebral extracellular (EC) potassium accumulation and an explanation model for the relationship between higher cerebral microdialysate (CMD) K^+^ concentration [K^+^] and cerebral metabolic distress and poor functional outcome, based on previously published literature (full line) and our findings (dashed line). For further description, see Discussion text.

The main findings of this study suggest that higher CMD [K^+^] levels are associated with brain metabolic distress and poor functional outcomes and that CMD [K^+^] levels do not correlate with corresponding plasma [K^+^] levels. The poor correlation between brain extracellular and plasma [K^+^] suggests integrity of the electrical barrier [16] and a primarily brain parenchymal origin of CMD [K^+^]. Brain extracellular K^+^ accumulation in the subcortical brain regions may be explained secondarily to membrane breakdown [3-5] and erythrocytolysis [2] (Figure 4). In line with that explanation, we found higher CMD [K^+^] in perilesional brain regions and an association with the presence of SAH related intraparenchymal hematoma. These findings suggest diffusion of extracellular K^+^ through the interstitial spaces with [K^+^] gradient from intracerebral hematoma and infarction to adjacent and more distant brain areas. Furthermore, although not statistically significant, almost all episodes of ICP >20 mmHg had CMD [K^+^] above the median, supporting the association between high CMD [K^+^] and cell damage. In this study, we may have missed transient brain extracellular K^+^ elevations because of our hourly sampling method, whereas a rapid-sampling microdialysis technique may detect rapid changes in brain extracellular [K^+^] [4].

Higher CMD [K^+^] was associated with elevated CMD lactate levels and brain metabolic distress. This association may be explained by the phenomenon of inverse neurovascular coupling triggered by excessive extracellular [K^+^], leading to vasoconstriction with consecutive regional hypoperfusion and metabolic derangement [25]. Moreover, excessive extracellular K^+^ has been recognized to play a pivotal role in the generation and propagation of cortical spreading depolarization (CSD) which, under a pathological environment, can lead to spreading ischemia [26-29]. Our study investigates CMD [K^+^] and cerebral metabolism of the subcortical white matter. Spreading depolarization as a cortical phenomenon is highly energy-demanding and has been shown by previous researchers to cause neuronal dysfunction in subcortical areas of the brain in rodent models [12,30-32]. In patients with aSAH, there is growing evidence that CSDs occur abundantly [33]. Investigators in a recent clinical study demonstrated that clustered CSDs are associated with a transient decrease in CMD glucose and an increase in CMD lactate and CMD LPR, which they measured by cerebral microdialysis [34] (Figure 4).

We found an independent association of both higher CMD [K^+^] and LPR with poor 3-month outcomes. Both parameters may be interpreted as surrogate markers for brain tissue injury after aSAH. According to this hypothesis, hemorrhage load and secondary brain injury are associated with disturbed brain metabolism and poor outcome [35-38] (Figure 4).

The potential toxic effect of K^+^ load in aSAH patients raises an important issue for neuroprotective strategies that may embrace buffering or release of excessive extracellular K^+^. Clinical management can rest on avoiding further K^+^ accumulation by maintaining balance between energy supply and demand in order to sustain the performance of the Na^+^/K^+^ pump for neuronal repolarization [39,40] and K^+^ clearance by glial cells [41].

Our study has several important limitations. (1) Only poor-grade aSAH patients observed at a single center are represented in this study. This may have introduced a selection bias restricting the generalizability of our data to the whole aSAH patient population. (2) No causative association between higher CMD [K^+^] and brain metabolism and patient outcome can be made on the basis of observational data. This study should be considered a pilot study conducted essentially to further elucidate pathophysiological mechanisms of secondary brain injury in patients with aSAH and to generate hypotheses based on our findings. (3) The low time resolution of K^+^ measurements in our study may have introduced a washout effect, which causes underestimation of peak [K^+^]. The perfusion fluid used for cerebral microdialysis contained K^+^ in its composition. Nevertheless, the high recovery rate for K^+^ found in the *in vitro* analysis suggested free diffusion of K^+^ with little obvious influence of the perfusion fluid K^+^. Therefore, CMD [K^+^] may be interpreted as an approximation of real [K^+^] of the brain extracellular space. (4) Our approach of taking samples daily for measurement cannot be used to assess the dynamics between brain ionic, metabolic and physiological homeostasis and simply focus on transversal relationships between these parameters.

Despite our study’s limitations, it addresses the association between brain extracellular [K^+^] with its systemic correlate and brain homeostasis in poor-grade aSAH patients. Moreover, our method proved to be feasible for bedside analysis of K^+^ in the severely injured brain. Future development and implementation of CMD point-of-care analytical methods seems to be necessary for a complete understanding pathophysiologic mechanisms of secondary brain injury after human aSAH.

## Conclusions

The findings of our pilot study suggest that higher CMD [K^+^] can be measured and may serve as a surrogate marker for brain injury in poor-grade aSAH patients. Further studies are needed to elucidate the relevance of interstitial K^+^ levels in the pathophysiology of secondary brain injury after aSAH.

## Key messages

• Higher K^+^ levels in the extracellular space of the subcortical white matter may serve as a biomarker for brain tissue injury in poor-grade aneurysmal subarachnoid hemorrhage patients.

• Work done in experimental models suggests that elevated K^+^ levels in the extracellular space can drive secondary brain injury after a subarachnoid hemorrhage. Further human studies are needed to confirm this hypothesis.

## Abbreviations

aSAH: Aneurysmal subarachnoid hemorrhage; CMD: Cerebral microdialysis; CPP: Cerebral perfusion pressure; CSD: Cortical spreading depolarization; CT: Computed tomography; GCS: Glasgow Coma Scale; ICP: Intracranial pressure; K^+^: Potassium; [K^+^]: Potassium concentration; LPR: Lactate/pyruvate ratio; mRS: Modified Rankin scale; P_bt_O_2_: Brain tissue oxygen tension.

## Competing interests

The authors declare that they have no competing interests.

## Authors’ contributions

APA and AJS were involved in the acquisition of data, statistical analysis, interpretation of data, study design and manuscript drafting. RH was involved in the study design, interpretation of data, statistical analysis, manuscript drafting and final revision of the manuscript. RB, BP, MF, AD, PL and ES participated in the acquisition and interpretation of data and in the final revision of the manuscript. FS and CT were involved in the study design and data acquisition and interpretation of the data. WOH and JPN were involved in the study design, data processing and statistical analysis. All authors read, critically reviewed and approved the final manuscript.
